# COVID-19 and Alzheimer’s disease: how one crisis worsens the other

**DOI:** 10.1186/s40035-021-00237-2

**Published:** 2021-04-30

**Authors:** Xiaohuan Xia, Yi Wang, Jialin Zheng

**Affiliations:** 1 Center for Translational Neurodegeneration and Regenerative Therapy, Tenth People’s Hospital of Tongji University, Shanghai, 200072 China; 2 Translational Research Institute of Brain and Brain-Like Intelligence, Shanghai Fourth People’s Hospital Affiliated to Tongji University School of Medicine, Shanghai, 200434 China; 3 Collaborative Innovation Center for Brain Science, Tongji University, Shanghai, 200092 China; 4 Department of Pharmacology and Experimental Neuroscience, University of Nebraska Medical Center, Omaha, NE 68198-5930 USA

**Keywords:** COVID-19, SARS-CoV-2, Alzheimer’s disease, Central nervous system, Inflammation, Cognitive impairment, Angiotensin converting enzyme 2

## Abstract

Alzheimer’s disease (AD) has emerged as a key comorbidity of coronavirus disease 2019 (COVID-19) caused by severe acute respiratory syndrome coronavirus-2 (SARS-CoV-2). The morbidity and mortality of COVID-19 are elevated in AD due to multiple pathological changes in AD patients such as the excessive expression of viral receptor angiotensin converting enzyme 2 and pro-inflammatory molecules, various AD complications including diabetes, lifestyle alterations in AD, and drug-drug interactions. Meanwhile, COVID-19 has also been reported to cause various neurologic symptoms including cognitive impairment that may ultimately result in AD, probably through the invasion of SARS-CoV-2 into the central nervous system, COVID-19-induced inflammation, long-term hospitalization and delirium, and post-COVID-19 syndrome. In addition, the COVID-19 crisis also worsens behavioral symptoms in uninfected AD patients and poses new challenges for AD prevention. In this review, we first introduce the symptoms and pathogenesis of COVID-19 and AD. Next, we provide a comprehensive discussion on the aggravating effects of AD on COVID-19 and the underlying mechanisms from molecular to social levels. We also highlight the influence of COVID-19 on cognitive function, and propose possible routes of viral invasion into the brain and potential mechanisms underlying the COVID-19-induced cognitive impairment. Last, we summarize the negative impacts of COVID-19 pandemic on uninfected AD patients and dementia prevention.

## Background

Coronavirus disease 2019 (COVID-19), caused by severe acute respiratory syndrome coronavirus-2 (SARS-CoV-2), is now considered a global public health emergency. SARS-CoV-2 predominantly attacks the human respiratory system and causes fever, colds, throat pains, coughs, dyspnea, and other respiratory symptoms [1]. In severe cases, acute respiratory distress syndrome (ARDS), pneumonia, acute cardiac problems, and multi-system organ failure (MSOF) have been observed [1]. Other atypical respiratory symptoms include headache, dizziness, anosmia, stroke, and deteriorated consciousness or memory [1]. Current evidence has supported that SARS-CoV-2 is capable of targeting and invading the central nervous system (CNS) [2]. In fact, neurological manifestations have occurred in more than one-third of COVID-19 patients with increased infection severity [3].

Alzheimer’s disease (AD) is one of the most common CNS comorbidities of COVID-19 [4]. AD is the most prevalent neurodegenerative disorder and the No.1 cause of dementia. In AD patients, brain regions responsible for memory and learning are damaged due to the deposition of amyloid beta (Aβ) or neurofibrillary tangles (NFT). Patients with moderate and severe AD are entirely dependent on caregivers. As COVID-19 is highly infectious and its management requires isolation and quarantine, the need of caregivers for AD management conflicts with that of COVID-19 [3, 4]. Therefore, COVID-19 adds extra burden on AD patients, caregivers, families, society, and the economy.

Identifying common etiological factors would provide new avenues for management and therapeutic strategies against both COVID-19 and AD. In this review, we link COVID-19 and AD by discussing the impacts of one on the other and summarizing the existing problems and potential strategies to improve the management of AD under the COVID-19 pandemic.

## COVID-19

### Epidemiology and symptoms of COVID-19

COVID-19 has spread worldwide and was declared as a pandemic by the World Health Organization (WHO) in March 2020. By March 24, 2021, a total of 123 902 242 confirmed cases of COVID-19 worldwide, including  2 727 837 deaths, have been recorded by the WHO (https://covid19.who.int/). To date, there have been 85 countries, territories and areas that have had more than 100 000 cases each, with the United States having the largest number of confirmed cases.

SARS-CoV-2 belongs to the family *Coronaviridae* and is an enveloped non-segmented, single-stranded, positive-sense RNA virus. SARS-CoV-2 can spread rapidly through various routes including droplets, aerosol, and fomite [5]. Moreover, fecal-oral and fecal-aerosol transmissions are suspected potential routes of SARS-CoV-2, as high viral loads have been detected in patients’ feces-related specimens [6, 7]. The majority of COVID-19 cases develop a lower respiratory tract infection, which leads to the high rate of viral transmission in densely populated areas or hospitals [8]. The median age of COVID-19 patients was reported to be 59 years (range, 15–89 years), and more than half of them were males [8]. People with low immune function, particularly the elderly and those with renal and hepatic dysfunction, are at a high risk of SARS-CoV-2 infection [8].

SARS-CoV-2 infection causes pneumonia with malaise, dry cough, fever and other symptoms such as lymphopenia, diarrhea, hemoptysis, headache, chills, muscle pain, sore throat, and loss of taste or smell. Generally, SARS-CoV-2 causes milder symptoms and lower case-fatality rate than severe acute respiratory syndrome coronavirus (SARS-CoV) or Middle East respiratory syndrome coronavirus (MERS-CoV), and most patients recover on their own [9, 10]. However, about 20% of the patients develop severe symptoms including acute lung injury, ARDS, septic shock, and multiple organ dysfunction syndrome, resulting in rapid death [9]. Besides the pulmonary disease, extra-pulmonary manifestations including neurological involvement have received increasing attention.

### Pathogenesis of COVID-19

**Fig. 1:**
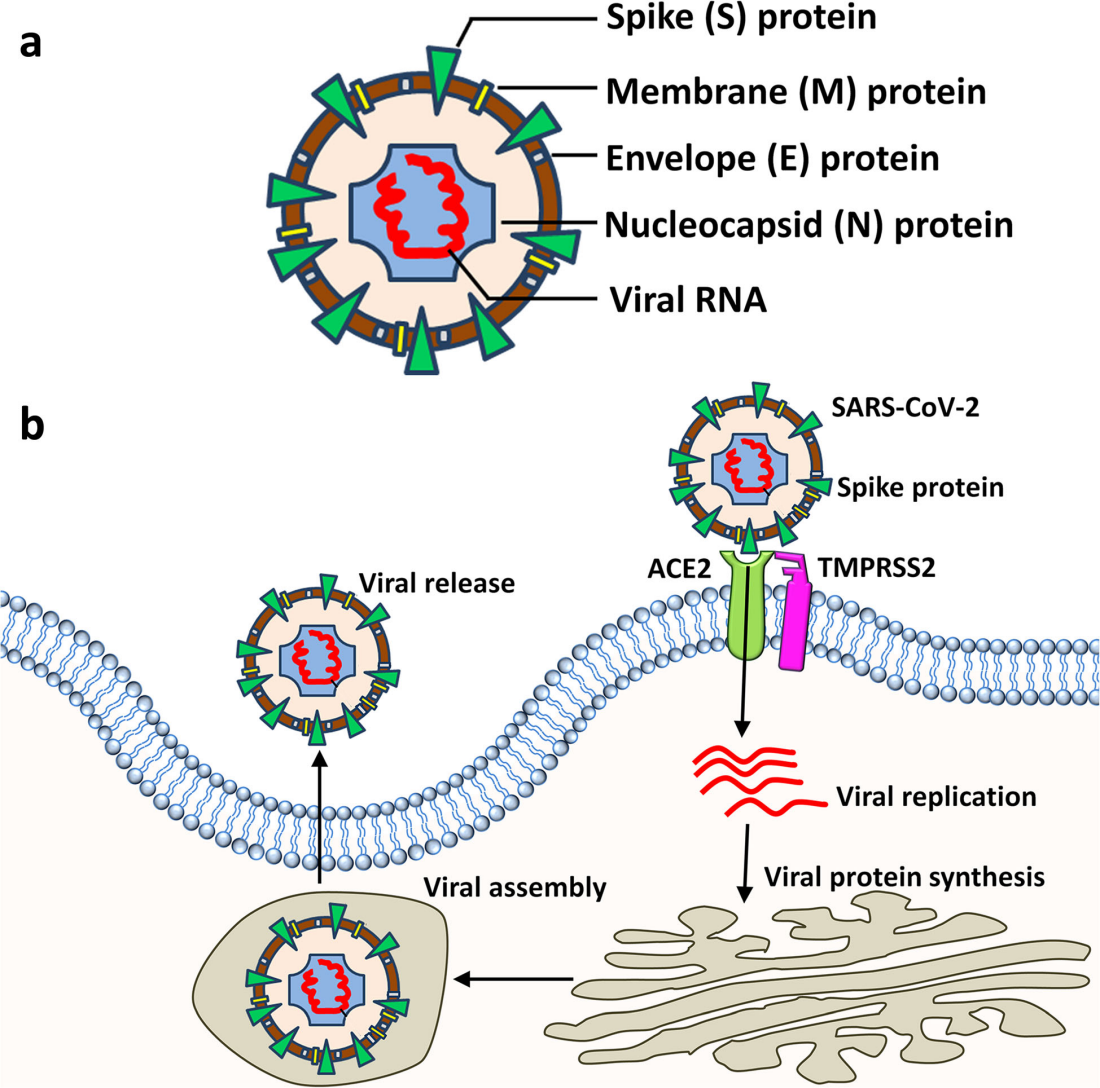
SARS-CoV-2 and viral infection. **a** A schematic of SARS-CoV-2 composition. A SARS-CoV-2 viral particle is composed of spike (S) protein, membrane (M) protein, envelope (E) protein, nucleocapside (N) protein, and viral RNA. **b** A schematic showing the infection of a host cell by SARS-CoV-2. With the aid of TMPRSS2, the S protein of SARS-CoV-2 binds with ACE2 to enter the host cell and uses the host machinery for translation and replication of the viral genetic material

SARS-CoV-2 enters the host alveoli through the respiratory tract. Inside the alveoli, SARS-CoV-2 infects type II pneumocytes by binding to its functional receptors. Currently, angiotensin converting enzyme 2 (ACE2) has been identified as the main mediator [11, 12]. ACE2 is a type I integral membrane protein of renin-angiotensin systems and is present on a variety of organs such as the heart, kidneys, and lungs. Spike (S) proteins of both SARS-CoV and SARS-CoV-2 form a trimer harboring a receptor-binding domain (RBD) that interacts with lysine 31 on ACE2 with high affinity [11, 13, 14]. Importantly, SARS-CoV-2 seems to recognize the human ACE2 more efficiently than SARS-CoV, which causes increased infection capacity of SARS-CoV-2 among people [14]. The interaction of the S protein of SARS-CoV-2 with ACE2 recruits the transmembrane protease serine 2 (TMPRSS2) to cleave the S protein at the S1/S2 site (C-terminal segment residues 697 to 716), enhancing viral entry [12, 15]. Other molecules can also mediate SARS-CoV-2 infection, since ACE2 knockout could not completely prevent SARS-CoV infection in rodents [16]. CD147 [17] and CD209L (L-SIGN) [18] have been identified as potential receptors for SARS-CoV-2, although more studies are needed to confirm their involvement (Fig. 1).

After being endocytosed into the cytoplasm, SARS-CoV-2 utilizes the host cell protein translation machinery to express the coronavirus polymerase complex, which is composed of non-structural proteins (Nsp) including RNA-dependent RNA polymerase (RdRp, Nsp12) and its co-factors (e.g., Nsp7, Nsp8) [19, 20]. RdRp further synthesizes a full-length negative-strand RNA template to make more viral genomic RNA and structural proteins [21–24]. The structural proteins, together with viral RNA, form mature SARS-CoV-2 that is released by exocytosis. This circle leads to uncontrolled viral replication, aggravating the course of COVID-19. Besides the type II alveolar epithelial cells, cardiomyocytes, vascular smooth muscle cells, renal tubular, and intestinal epithelial cells are also predicted to be targets of SARS-CoV-2, as the co-expression of ACE2 and TMPRSS2 has been identified in these cells by single-cell RNA-sequencing analyses [25, 26]. This explains the pathophysiology of acute lung and myocardial injury, and gastrointestinal symptoms reported in COVID-19 cases.

The budding off of SARS-CoV-2 leads to pyroptosis, an inflammatory form of programmed cell death that stimulates alveolar macrophages [27]. The activated macrophages release pro-inflammatory cytokines and chemokines including interleukin-1 (IL-1), interleukin-6 (IL-6), tumor necrosis factor-alpha (TNF-α), interferon-γ, MCP-1/CCL2, macrophage inflammatory proteins, and C-X-C motif chemokine ligand 10 [28]. These molecules can cause smooth muscle dilation and contraction of blood vessel endothelial cells, negatively influencing the capillary permeability. The rapid increase of cytokines and chemokines causes recruitment of more immune cells (such as T cells, neutrophils and monocytes) in the blood to the lung tissue [29]. The accumulation of immune cells leads to hyperinflammation and cytokine storm that attack more healthy cells. The leakage of plasma from blood vessels into the interstitial spaces and the destruction of lung parenchyma that reduces surfactant production and alveolus surface tension result in alveolar collapse and alveolar edema. Consequently, gas exchange is impaired and refractory hypoxemia is formed, resulting in ARDS.

Moreover, the excessive vascular cytokines can be taken up by other tissues and trigger the systemic inflammatory response syndrome [30]. Once reaching the CNS, especially the hypothalamus, the excessive IL-1 and IL-6 may increase the production of prostaglandins, which elevates the core body temperature to initiate fever. The vascular cytokines also induce capillary hyperpermeability, causing the deposition of plasma within various tissues to decrease the total peripheral resistance and blood pressure, resulting in exhausted perfusion [30]. The decreased perfusion further increases the blood urea nitrogen and creatinine in the kidney, leading to acute renal injury [31]. In addition, disseminated intravascular coagulation (DIC) has been reported as a severe complication in some COVID-19 cases [32]. ARDS, severe renal injury and DIC, together with other complications including cardiac dysfunction in COVID-19 [1], ultimately lead to MSOF and death.

### AD

**Fig. 2:**
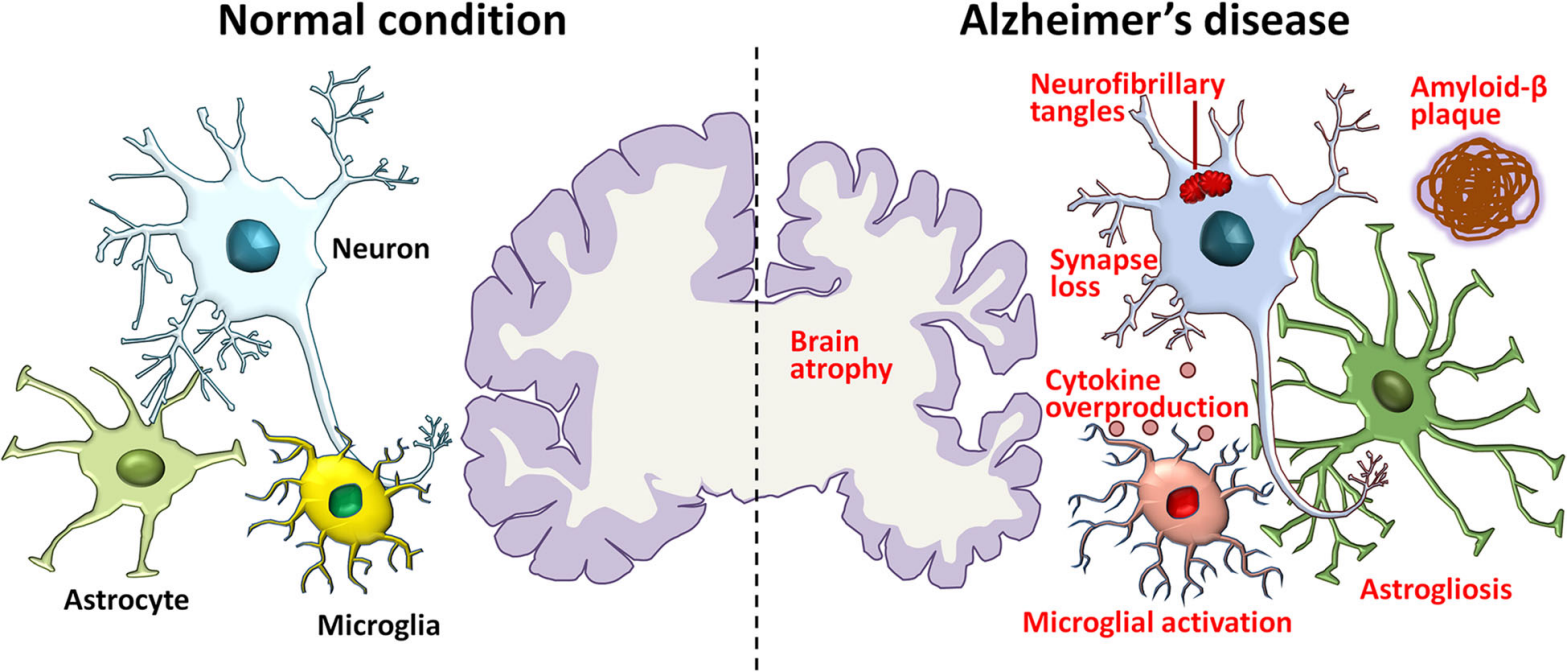
Pathological hallmarks of AD. A schematic of the pathological changes in AD brains compared with normal brains. Brain atrophy resulting from neuronal loss is recognized at the gross anatomical level. Accumulation of toxic amyloid-β, formation of intraneuronal neurofibrillary tangles, loss of synapses, activation of microglia, astrogliosis, overproduction of cytokines, and dystrophy of neurites are observed at the microscopic level

AD is the No.1 cause for dementia and is a global public health priority with no effective treatments. The lack of effective treatment is largely due to its unclear pathogenesis. Several hypotheses have been raised to elucidate the mechanism of AD (Fig. 2).

### Aβ

The Aβ hypothesis suggests that the accumulation of Aβ_40–42_ produced through sequential cleavage of β-amyloid precursor protein (APP) by β- or γ-secretase in the brain leads to the formation of senile plaques, which represents the primary pathological process. The Aβ hypothesis is supported by findings from genetics: (1) familial AD mutations of *APP* and *PSEN1/2*, transcripts encoding two key components of the γ-secretase complex, are involved in either Aβ generation or Aβ processing and result in excessive production of Aβ_40–42_ [33]; conversely, an APP missense mutation (A673T) results in a lifelong decrease in APP cleavage by β-secretase, offering a reduced clinical risk of AD [34]; (2) the epsilon4 allele of apolipoprotein E (*ApoE*) is the major genetic risk factor for sporadic AD, highly likely through the effects on Aβ metabolism [35].

Interestingly, recent studies have indicated that the soluble Aβ oligomers are more toxic than fibrillar Aβ accumulated in dense plaques. The Aβ oligomers purified from AD brains can inhibit long-term potentiation and cause synaptic dysfunction, dendritic spine damage, and neuronal death of cultured neurons [36]. In addition, Aβ oligomers can induce hyperphosphorylation of tau and the formation of NFT [37]. Therefore, plaques can serve as the “reservoirs” for soluble Aβ oligomers to exert downstream pathological events.

### Tau

NFT that is mainly composed of hyperphosphorylated tau is another hallmark of AD. In parallel with NFT formation, neuronal and synaptic loss occur, so the clinical features and the severity of AD are usually closely correlated with the NFT pathology [38]. Tau hyperphosphorylation and NFT formation usually occur following the formation of senile plaques, suggesting that NFT is a downstream cascade of Aβ accumulation [37]. However, recent studies have indicated that the tau events act independently as *tau* gene mutations can cause frontotemporal dementia (FTD) without Aβ plaques [39]. This suggests that tau and Aβ may work in parallel to enhance each other’s toxicity to drive AD.

While Aβ and NFT have served as promising hypotheses to explain AD, post-mortem examination of these classic pathological hallmarks only explains to a limited degree the levels of AD and dementia in the population [40]. In addition, all clinical trials with strategies targeting Aβ or NFT have been unsuccessful so far. Therefore, other alternative hypotheses may explain the pathogenic mechanisms of AD and help design innovative interventional strategies.

### Neuroinflammation

The neuroinflammation hypothesis of AD was first proposed after the identification of immune elements in senile plaques. Although neuroinflammation has been widely considered to play a key role in the pathophysiology of AD, what remains unclear is whether neuroinflammation acts as the underlying cause, or the consequence, of the disease. Recent evidence has suggested that neuroinflammation contributes to the AD pathogenesis to an extent no less (if not more) than Aβ or tau, instead of being a passive system activated by accumulated senile plaques or NFT [41]. The strongest supporting evidence is that immune receptors, like triggering receptors expressed on myeloid cells 2 protein [42] and CD33 [43], are closely associated with AD.

Microglia exhibit a heightened activity state in AD, characterized by increased production and release of cytokines and reactive oxygen species [44]. Microglia can recognize and bind to soluble Aβ oligomers and aggregated Aβ *via* cell-surface receptors, including multiple CD family members and Toll-like receptors (TLRs) [41]. Aβ binding activates microglia, leading to the production of proinflammatory molecules. Genetic deletion of *CD36*, *TLR4*, or *TLR6* in vitro decreases the Aβ-induced cytokine production, and terminates cellular Aβ aggregation and the resulting inflammasome activation [45]. Astrogliosis usually occurs following microglial activation, aggravating neuroinflammation [46]. Other cells like blood-derived mononuclear cells, are also a part of the neuroinflammation hypothesis of AD [47].

### Viral infection

Numerous studies have associated AD with viral pathogens. Comprehensive molecular profiling in a large patient cohort has identified multiple viruses that have an impact on AD-associated biology [48]. Among them, the herpesvirus family including herpes simplex virus (HSV) and human herpesvirus (HHV) is the most studied viral family for AD [48–52]. The hypothesis that considers HSV-1 as a causative factor for the development of AD can be tracked back to 1982 [53]. To date, accumulating results from epidemiological, post-mortem, animal, and cell-culture studies have supported this hypothesis [54]. For example, results from a population-based cohort study showed that the reactivation of HSV seropositivity is highly correlated with incident AD [55]. HSV-induced herpes simplex encephalitis (HSE) causes damage to the hippocampus as well as to the temporal and frontal lobes, same brain areas that are affected in AD, and induces a cognitive phenotype similar to AD [56, 57]. HSV-1 DNA has also been shown to specifically co-localize with AD pathology in the brains of AD patients [58]. Moreover, cognitive impairment has been observed together with neuroinflammation in infected cortical and brain stem regions in HSE mice [59]. Further studies have demonstrated alterations of Aβ metabolism, dysregulation of calcium homeostasis, synaptic dysfunction, and apoptosis in cultured human neuronal and glial cells infected with HSV-1 [60]. In addition, anti-HSV drugs have been shown to reduce Aβ and p-tau accumulation in the infected mouse brains [61]. All these findings suggest the strong association of HSV-1 with the pathogenesis of AD. Besides HSV-1, multiscale analysis of three independent Alzheimer’s cohorts carried out by Readhead et al. identified increased human herpesvirus 6A (HHV-6A) and human herpesvirus 7 in subjects with AD compared with controls, and demonstrated regulatory relationships linking viral abundance and modulators of Aβ production [48]. Both in vitro and in vivo studies have further shown that HHV-6A infection induces the expression of Aβ, the phosphorylation of tau protein, and the activation of microglial cells [48, 51, 52]. The pathological effects of HHV-6A on AD are very likely caused by the suppression of miR-155 and dysregulation of the autophagy/unfolded protein response interplay [48, 51]. Another animal study revealed that infection of 3xTg-AD mice with mouse hepatitis virus exaggerated tau pathology and accelerated AD progression [62]. These studies provide compelling evidence for the contribution of specific viral species to the development of neuropathology and AD.

In summary, Aβ and NFT are the best-known hypotheses of AD, although they do not entirely elucidate the pathogenic mechanism. Recent evidence has supported for the neuroinflammation hypothesis, though the causality needs to be addressed. Emerging evidence has also implicated viral infection in AD, although future studies are needed to identify the underlying mechanisms for how each specific viral infection leads to AD.

## AD increases COVID-19 morbidity and mortality

Clinicians and scientists have made great efforts in identifying the potential risk factors for COVID-19, and they point out that AD is strongly linked with the morbidity and mortality of COVID-19.

### AD patients exhibit elevated morbidity and mortality of COVID-19

Emerging evidence has suggested that AD significantly increases the morbidity of COVID-19. A retrospective study in Spain has suggested cognitive impairment (29.1%) as one of the most frequent comorbidities in deceased COVID-19 patients [63]. Moreover, AD was the most common diagnosis for cognitive impairment in the confirmed COVID-19 group (9.3% of all patients) [63]. More importantly, another observational study in Spain reported a higher proportion of COVID-19-infected cases in the AD population (15.1%) [64]. Given this evidence, AD appears to be an important influential comorbid of COVID-19.

Multiple studies have claimed AD as a key contributor to COVID-19 mortality. Data collected from a tertiary hospital in Spain showed that subjects with cognitive impairment had shorter survival from the onset of symptoms than patients without cognitive impairment [63]. Furthermore, significantly higher death rates were observed in COVID-19 patients with AD (54.5%), than in patients with FTD (*χ*^2^ = 4.94, *P* = 0.045), suggesting that the progression of AD correlates with the severity of COVID-19 [64]. In addition, a Korean team has evaluated the contribution of various factors, such as age, AD, chronic lung disease, stroke, hypertension, coronary vascular disease, dyslipidemia, chronic kidney disease, diabetes, and history of taking angiotensin II receptor blockers or ACE inhibitors, to the death rate of COVID-19 patients in a multicenter retrospective cohort, using multivariate logistic analysis, and found that only age, AD, and chronic lung disease are significant parameters for predicting COVID-19 non-survival (*P* < 0.05) [65]. In addition, data on COVID-19 Case Mortality Rates (CMR) in 93 countries have demonstrated that AD has higher positive correlations with CMR (*r* = 0.36), compared with respiratory diseases including asthma (*r* = 0.28) and chronic obstructive pulmonary disease (*r* = 0.27) [66]. These findings implicate AD as an essential population risk factor for COVID-19 mortality.

### The potential contributing factors to the AD-mediated elevation of COVID-19 morbidity and mortality

#### Aging

Aging is the No. 1 risk factor for AD. AD generally (in about 90% of cases) affects individuals over 65 years and its prevalence doubles every 5 years, generating a time-dependent exponential increase [67]. Aging is also a prominent risk factor for severe disease and death from COVID-19 [68, 69]. COVID-19 patients over 59 years are at least 5 times more likely to die after development of symptoms than those below 59 in Wuhan, China [69]. Similarly, the case fatality ratio (CFR) of COVID-19 in the 80s and above is about 2-fold higher than the overall CFR in Italy, the first country affected by the pandemic after China [70]. Aging may induce production of reactive oxygen species, modification of epigenetics, and alterations of gene expression or non-coding RNA expression levels, which contribute to the pathogenesis of both AD and COVID-19 [71, 72].

#### Lack of self-care and cognitive abilities

The elevated COVID-19 morbidity in AD patients may be partially due to their distinct living states. AD patients lack the ability to care for themselves and have a reduced awareness of environmental changes. Therefore, a great proportion of AD patients are living in nursing homes, which, however, were attacked most heavily by the COVID-19 epidemic in many countries. For instance, nursing home residents accounted for 40% of all deaths in the United States during the first wave of COVID-19 epidemic, although the nursing home population may be less than 1% of the whole population [73]. Being a respiratory virus, SARS-CoV-2 can propagate rapidly among individuals who live in the same location or work in industries with high degree of interpersonal closeness [73]. The nursing homes have high population density and large household size, providing a perfect environment for SARS-CoV-2 transmission among AD patients.

The lack of awareness of environmental changes also hinders AD patients from self-protection. An investigation in Japan reported that the rate of recognition of the COVID-19 pandemic was only 38.2% in AD patients [74]. Moreover, 74.5% of the AD patients failed to wear face masks properly by themselves, likely due to the cognitive impairment that further limited their recognition of the pandemic. Consistently, another survey in Japan revealed that only 31% and 24% of AD patients were aware of the COVID-19 epidemic and the reason for wearing a mask, respectively [75]. The lack of awareness on maintaining “social distance” (1.5–2 m) or wearing face masks may increase the infection risk of SARS-CoV-2 in AD patients. AD patients also have difficulties in remembering safeguard procedures, which further increases their risk of infection.

#### Direct pathologic changes of AD

*ApoE* is the strongest risk gene for sporadic AD [35], and may be involved in SARS-CoV-2 infection. The *ApoE* e4 allele has been predicted to increase the risk of severe SARS-CoV-2 infection in the UK Biobank community cohort [76]. Protein products of the *ApoE* cluster genes may act as SARS-CoV-2 receptors since they have been proved to be receptors for various viruses including hepatitis virus C and herpesvirus [77]. Moreover, ApoE dysfunction is associated with cardiovascular diseases and obesity that increase the vulnerability to COVID-19 [78, 79].

AD may facilitate SARS-CoV-2 infection through Ca^2+^ dysregulation. Aβ oligomers integrate into the plasma membrane and form pores, allowing the passage of Ca^2+^ [80]. Aβ oligomers also stimulate *N*-methyl-*D*-aspartic acid receptor, α-amino-3-hydroxy-5-methyl-4-isoxazole-propionicacid receptor, and L-type voltage-gated calcium channels directly, leading to an increase in intracellular Ca^2+^ [81]. The abnormally increased intracellular Ca^2+^ contributes to AD through multiple mechanisms including the formation of NFT, electrophysiological disorder, and neuronal death/degeneration [82]. SARS-CoV and MERS-CoV both utilize Ca^2+^ for viral infection [83]. Furthermore, various RNA viruses alter Ca^2+^ homeostasis through the disruption of calcium channels and pumps, resulting in host cell death that benefits viral replication [84]. Thus, despite the lack of sufficient experimental data, it is possible that the Ca^2+^ dysregulation in AD brains facilitates the life cycle of viruses, including the spread of viral infection in the COVID-19 epidemic.

Additionally, a genome-wide association study found that the *Ace2* gene, but not the *Ace1* gene, had higher expression levels in brain tissues from AD mice than in control samples [85]. In contrast, *Ace2* gene expression levels in the peripheral blood did not differ in healthy versus diseased brain tissues. Similar trends were observed in human brain tissues. Microarray analysis showed that the expression levels of *Ace2* increase with the severity of AD [85, 86]. In contrast, *Ace1* transcript levels were reduced in brain tissues from AD patients with mild cognitive impairment, compared with healthy donors. The excessive production of ACE2 in AD brains may facilitate the invasion of SARS-CoV-2 into the CNS and accelerate viral transmission. In addition, ACE2 can hydrolyze Ang II to produce Ang-(1–7), and Ang-(1–7) further binds with its receptor Mas to regulate various downstream signaling cascades including the PI3K/Akt/CREB/BDNF/TrKB pathway [87, 88]. These findings imply a role of ACE2 in regulating the neurological and mental outcomes of SARS-CoV-2 infection, which needs to be confirmed in future studies [89]. In addition, recent studies have indicated that the reduced activity of the ACE2/Ang(1–7)/Mas axis is largely linked to tau hyperphosphorylation and accumulation of neural internal microtubules and Aβ peptides [90, 91]. Based on the above evidence, it can be hypothesized that SARS-CoV-2 may target ACE2 and repress its expression or activity, causing disturbances in cognitive function and exacerbating cognitive impairment in people with AD, compared with healthy controls and patients with vascular dementia.

Acetylcholine (Ach) is an excitatory neurotransmitter that is essential for memory and learning. Activities of choline acetyltransferase (ChAT), the enzyme responsible for Ach synthesis, are significantly reduced, most severely in the temporal lobe, in AD patients only [92]. Choline uptake and Ach release decrease due to the decline of ChAT activity, causing dramatic presynaptic cholinergic impairment [93]. Thus, major brain functions including memory, learning, waking, and sleep are impaired in AD [93]. The involvement of Ach in COVID-19-driven inflammation has been implied by an in silico study [94], which showed that higher prenatal choline levels in the mother’s body can protect the developing brain of the fetus from the adverse effects of SARS-CoV-2 infection. According to these findings, the loss of Ach production in the CNS in AD patients may eliminate an important preventive mechanism against inflammation, contributing to the uncontrolled cytokine storm in COVID-19.

Another key pathological feature of AD is neuroinflammation. The Alzheimer’s brain contains elevated microglial-derived cytokines (e.g., TNF-α, IL-1β, and IL-6) and other immune mediators, reflecting a chronic inflammatory microenvironment [95]. Similarly, serum TNF-α and IL-6 levels have been reported to be significantly higher in AD patients than in healthy donors [96]. As discussed above, COVID-19 induces systemic inflammatory responses and cytokine storm in severe cases [3]. The dramatically elevated cytokine levels in patient serum have been closely correlated with COVID-19 fatality [97]. Plasma proteomics profiling has also identified cytokines/chemokines to be most perturbed in COVID-19 patients and implicated them as early biomarkers to monitor disease severity [72]. Based on this observation, AD and COVID-19 have similar inflammatory signaling. The existing inflammation in AD patients may accelerate the accumulation of pro-inflammatory cytokines post SARS-CoV-2 infection, exacerbating the immune responses and increasing the mortality of COVID-19.

#### Comorbidities of AD

Recently, the medical comorbidity of AD has received much attention, since the single-disease framework may weaken the therapeutic effects due to a lack of consideration of multimorbidity. Hypertension (55%), osteoarthritis (38%), depression (32%), diabetes mellitus (26%), and coronary artery disease (23%) are five most common comorbidities in AD [98]. Moreover, a comparative study has also identified that the occurrence of morbidities of diabetes mellitus and depression in AD patients is significantly higher than that in controls, suggesting the strong association of AD with these two illnesses [84]. Diabetes has been reported as a high-risk factor for COVID-19 [99]. In a retrospective study employing multi-center cohorts in China, diabetes has been identified as the second most common (19%) comorbidity in COVID-19 patients [100]. In deceased patients, the percentage of diabetes comorbidity soared to 31%. A whole-population study carried out in England also found that diabetes was associated with significantly increased odds of COVID-19 mortality [101]. Diabetes has been proposed to increase the in-hospital death of COVID-19 patients *via* cardiovascular events, thromboembolism, and DIC [102]. In addition, hypertension and coronary heart disease are common comorbidities of both AD and COVID-19 [98, 100]. However, whether AD could increase the risk of hypertension and coronary heart disease remains controversial, since physicians tended to underdiagnose other illnesses in AD patients due to the inability of the patients to describe or remember specific complaints and to cooperate with extensive diagnostic tests [103]. Overall, these clinical studies reveal that AD comorbidities, especially diabetes, are associated with poor outcomes of COVID-19.

#### Drug-drug interactions

Currently, cholinesterase inhibitors (ChEIs) (e.g., donepezil, rivastigmine and galantamine) are widely used for AD treatment [104]. The ChEIs can be affected by substracts, inhibitors or inducers of the cytochrome P450 (CYP450) enzymes (e.g., CYP2D6, CYP3A4) [105]. Potential drugs for COVID-19 such as chloroquine (CQ) and hydroxychloroquine (HCQ) can be metabolized by CYP2D6 and CYP3A4, which may lead to significant alterations of the pharmacological effects of ChEIs [106]. A similar situation may be observed when azithromycin, another potential drug for COVID-19, is used with AD treatment. Besides, the anti-viral protease inhibitor lopinavir/ritonavir inhibits CYP3A and CYP2D6, activates CYP1A2, CYP2B6, CYP2C19, CYP2C9 and glucuronyl transferase enzymes, and suppresses drug transporters like glycoprotein [107]. Lopinavir/ritonavir may increase the plasma concentrations of ChEIs, resulting in adverse reactions or toxicity risk [106]. Thus, cardiac adverse effects (such as bradycardia, heart block, and QT interval prolongation) may appear in relation to both ChEIs and CQ/HCQ or azithromycin, which ultimately elevates the death rate of COVID-19 [106].

#### Nutritional disorders

Malnutrition has been identified with high prevalence in AD patients of different severity. A study carried out in Italy reported that over 95% of AD patients were in a malnourished condition or were at high risk of malnutrition [108]. In a cross-sectional study, over one-fourth of the COVID-19 patients were at risk of malnutrition and more than half of the COVID-19 patients were in a state of malnutrition in China [109]. Malnutrition is associated with the length of hospital stay and the in-hospital mortality [110, 111]. For instance, the incidence of diabetes and other comorbidities is also significantly altered in COVID-19 patients with risk of malnutrition or in malnutrition [109]. Therefore, malnutrition caused by AD may negatively affect the prognosis of COVID-19.

In summary, growing evidence has implicated AD in the pathogenesis of COVID-19. Individuals with AD present pathological abnormalities from molecular to system levels, together with lifestyle alterations. All these changes would in turn dramatically increase the morbidity and mortality of COVID-19 *via* increasing the infection risk, facilitating viral infection, worsening patient conditions, and accelerating patient death. Therefore, more intensive care is urgently needed to improve the situation of AD patients in the COVID-19 crisis and specialized/personalized therapeutic strategies are required in the treatment of COVID-19 patients with AD.

## COVID-19 may promote the initiation and progression of AD

 It is increasingly evident that COVID-19 may lead to neurological symptoms [3], which include, but are not limited to, dizziness, headache, impaired consciousness, and seizure. Besides acute neurological issues, long-term neurological consequences of COVID-19 including AD have emerged as an area of concern.

### COVID-19 contributes to cognitive impairment

The negative effects of COVID-19 on cognitive function have been demonstrated by studies worldwide. An observational study in China demonstrated that 14.8% of COVID-19 patients suffered from impaired consciousness [3]. Similarly, a relevant proportion of patients after critical SARS-CoV-2 infections show memory and executive function deficits in Europe [112, 113]. In an observational study, 65% of patients were noted to have confusion according to the Confusion Assessment Method for the intensive care unit (ICU) [113]. At discharge, 15 of 45 (33%) patients still had cognitive and motor deficits, including inattention, disorientation, or poorly organized movements in response to command. This is consistent with existing data that long-term cognitive impairment is commonly present in ARDS survivors at discharge [114]. Therefore, cognitive deficits commonly occur in COVID-19 patients, who may develop AD as a long-term consequence.

### Potential routes for SARS-CoV-2 invasion into the CNS

**Fig. 3:**
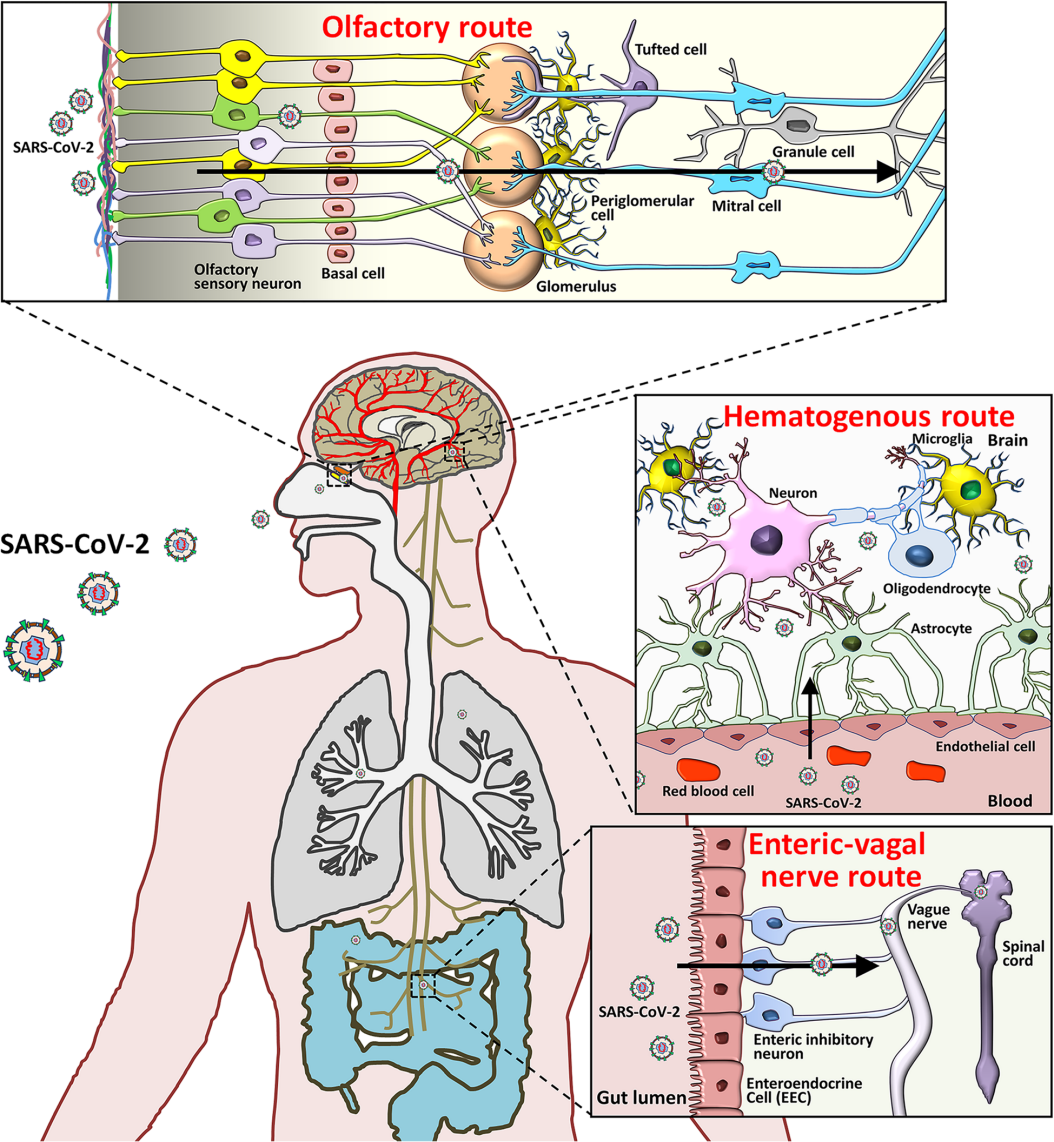
Potential routes of SARS-CoV-2 invasion into the CNS. Based on the tissue/cell expression patterns of the viral binding receptor ACE2 and cell entry-associated proteases TMPRSS2 on the olfactory epithelium, myelin-forming cells, enteric neurons, and vascular endothelium, it is plausible that SARS-CoV-2 enters the human brain *via* the neural route of myeline sheaths of olfactory, enteric and vagal nerves, and *via* the hematogenous route across the blood-brain barrier

Multiple studies have independently demonstrated CNS infection of SARS-CoV-2 in postmortem brain tissues of COVID-19 patients, animal models, and cultured cells [115, 116]. SARS-CoV-2 may enter the CNS either through the indirect hematogenous route or the direct neural route (Fig. 3) [117]. Viral RNA has been detected in the peripheral blood specimens collected from COVID-19 patients on the first 2 to 3 days after the onset of symptoms [118]. The presence of SARS-CoV-2 in blood allows it to pass into the cerebral circulation, where the slow blood flow may facilitate the interaction of SARS-CoV-2 S protein with ACE2 expressed on the capillary endothelium [117]. Subsequent budding of viral particles from the capillary endothelium leads to endothelial lining damage, which may further aid viral access to the brain [117]. This premise has been confirmed in an advanced 3D microfluidic model of the human blood-brain barrier [119]. In this model, the SARS-CoV-2 protein triggers a pro-inflammatory response on brain endothelial cells and promotes loss of barrier integrity, which can assist the invasion of SARS-CoV-2 to the brain. In vitro studies have further indicated that, once within the neuronal tissues, SARS-CoV-2 interacts with ACE2 and infects neurons [115, 120]. Treatment with ACE2 antibodies or cerebrospinal fluid from a COVID-19 patient significantly inhibited the viral infection of neurons [115]. Importantly, before the occurrence of the proposed neuronal damage, endothelial ruptures in cerebral capillaries and bleeding within cerebral tissue may lead to fatal consequences in patients with SARS-CoV-2 infections [117].

The hematogenous route can explain cerebrovascular symptoms, but falls short of explaining neurological symtpoms like olfactory or gustatory dysfunctions that, in some COVID-19 cases, occur even before repiratory symptoms [121]. Previous studies have demonstrated the invasion of SARS-CoV to the brain of a transgenic mouse expressing human ACE2 *via* nose close to the olfactory epithelium [122]. Similarly, SARS-CoV-2 has also been reported to infect the brain tissues of transgenic mice with human ACE2 expression by intranasal administration, supporting the hypothesized olfactory route for SARS-CoV-2 entry to the human brain [123]. The findings that ACE2 and TMPRSS2 are expressed in olfactory sensory neurons, olfactory epithelial supporting and stem cells [124], and CNS cells [120], further support neural invasion by SARS-CoV-2 through the olfactory route. SARS-CoV-2 also appears to be able to enter the CNS *via* vagus and enteric nerves as ACE2 and TMPRSS2 expression has been confirmed in enteric neurons and glia [125]. In human intestinal organoids, SARS-CoV-2 can infect and replicate in enterocytes [126]. Therefore, the spread of SARS-CoV-2 in the CNS through the vagus nerve that synapses to enteric neurons seems to be another invasion mechanism. To summarize, SARS-CoV-2 may invade the CNS through hematogenous and neural (olfactory and enteric-vagal) routes.

### Potential mechanisms of COVID-19-mediated cognitive impairment

COVID-19 affects cognitive function in complex ways. First, studies have suggested that viral infection per se increases the risk of cognitive impairment directly or indirectly [117]. Second, COVID-19 management poses greater risk of cognitive impairment on individuals due to the required isolation. In this section we discuss the potential mechanims through which the COVID-19 pandemic induces cognitive impairment.

#### Viral infection

SARS-CoV-2 presents neurotropism and is likely to be able to cause cognitive impairment directly in a similar way to SARS-CoV [117]. However, at present, there is no solid evidence showing direct induction of cognitive dysfunction by SARS-CoV-2 infection. Thus, scientists speculate that the virus probably exerts its effects on cognition *via* indirect pathways. First, the virus may affect cognition by attacking the cerebral vascular system. Varga et al. have reported that SARS-CoV-2 produces endothelitis, which is a root cause for several pathological conditions like cerebral ischemia [127]. Large-vessel stroke is another cerebral vascular disease that may be directly attributed to viral infection in people, especially young people, infected with SARS-CoV-2 [128]. A case report has also demonstrated the occurrence of small asymptomatic ischemic strokes in COVID-19 patients hospitalized in an ICU in France [113]. These results indicate a correlation between SARS-CoV-2 infection and impaired brain function; however, the causality remains to be determined.

Previous studies on other human coronaviruses may shed light on this question. Animal studies on human coronavirus OC43 have revealed that the hippocampus (especially CA1 and CA3 regions), which controls memory and cognition, seems particularly vulnerable to coronavirus infection [129, 130]. This hippocampal vulnerability is highly likely to cause negative effects on learning and memory. However, more studies are required to address the questions of whether human coronavirus infection leads to the similar hippocampal-related degeneration as in AD and accelerates disease onset in previously unaffected individuals.

#### Inflammation

A great proportion of COVID-19 patients show a severe innate immune response and sustained rise of systemic cytokine levels. The systemic inflammation has been demonstrated to impact cognitive function and promote progression of neurodegenerative diseases [41]. For example, human cognitive performance has been inversely correlated with chronic peripheral elevation of IL-6 [131]. Similarly, increased IL-1β in rodent brains results in impairment of long-term potentiation and cognitive performance, together with elevated Aβ and NFT production [132]. Furthermore, knockout or blockade of proinflammatory cytokines such as IL-6 and IL-1 improves reference and spatial memory and causes better cognitive performance as ascertained by multiple memory and learning behavioral tests [133, 134]. In addition, COVID-19 may induce the activation of the NOD-, LRR- and pyrin domain-containing protein 3 (NLRP3) inflammasome, which plays a key role in the development of ARDS [135]. After being activated, the NLRP3 inflammasome exerts adverse effects on the normal phagocytic function of microglia, leading to the failure of Aβ clearence in the brain [136]. Therefore, COVID-19-induced inflammation, especially the cytokine storm, may directly lead to cognitive dysfunction and neurodegeneration, and COVID-19 survivors may experience AD in the following years.

#### Potential APP metabolism dysfunction

Surprisingly, several recent multi-omic analyses of samples from COVID-19 patients have revealed a potential relationship between COVID-19 and APP metabolism. For example, RNA-seq analysis has demonstrated a significant increase of *APP* transcript in the blood samples from COVID-19-positive patients, compared with COVID-19-negative ones in the United States [137]. Furthermore, a single-cell RNA-seq study carried out by Yang and colleagues has identified *APP* as one of the most up-regulated genes in oligodendrocytes isolated from the post-mortem brain tissues of COVID-19 patients [138]. However, it is worth noting that the dysregulation of APP metabolism-related genes in COVID-19 patients is not constantly observed among multi-omic studies. Multi-transcriptome sequencing in the red blood cell-depleted whole blood failed to identify any difference in the expression levels of *APP* and *PSEN1/2* between COVID-19 patients and uninfected donors in China [139]. Similarly, no siginificant difference was observed in the levels of transcripts of *APP* and *PSEN1/2* between SARS-CoV-2-infected and uninfected human pluripotent stem cell (hPSC)-derived brain organoids [140]. Thus, whether or not COVID-19 influences APP metabolism remains an open question that needs to be addressed in the future.

#### Long-term hospitalization and delirium

It has been reported that neurological issues have occurred in up to 20% of COVID-19 patients who require ICU admission [3]. Multiple symptoms, such as anxiety, depression, prolonged pain, impaired cognitive and physical function that are known as post-intensive care syndrome, commonly occur in ICU patients. In ARDS that is not caused by COVID-19, up to 70%–100% of individuals show cognitive problems such as impaired executive function, short-term memory deficit and anxiety at discharge [114]. At 1 or 2 years post-hospital discharge, the proportions of ARDS survivors with cognitive impairment remain at around 50% [114]. Around 20% of ARDS survivors suffer from cognitive deficits like impaired executive function, short-term memory deficit, and anxiety at 5 years [114]. More importantly, animal studies have demonstrated that isolation or restraint stress that mimicks long-term hospitalization and delirium, significantly promotes amyloid plaque formation and tau phosphorylation in AD mice [141, 142]. These observations indicate that patients who require long-term hospitalization or recover from COVID-19 after leaving ICU are potentially at higher risk of long-term residual neurocognitive conditions, which may play a significant role in the development of AD after critical COVID-19.

#### Post-COVID-19 syndrome

COVID-19 may cause long-term adverse effects, similar to SARS [143]. After an acute SARS episode, many patients develop chronic fatigue syndrome/myalgic encephalomyelitis, sickness syndrome manifestations (malaise, anorexia, fatigue, and myalgia), endocrinopathy, long-term psychiatric conditions such as post-traumatic stress disorder (PTSD), and other syndromes. These are collectively called the “post-SARS syndrome” [143–145]. It is hypothesized that COVID-19 survivors may experience similar long-term adverse effects as those with post-SARS syndrome [143]. To date, post-COVID-19 paediatric inflammatory syndrome, Guillain-Barré syndrome, depression, and PTSD have been reported by various independent studies [146]. Unfortunately, apart from the chronic inflammation discussed above, other symptoms that are observed in post-COVID-19 syndrome may also be linked with AD. For instance, Guillain-Barré syndrome cases present with increased serum levels of transforming growth factor-β1 (TGF-β1) [147]. An abnormal increase of TGF-β1 in the brains of AD patients has also been detected, which may be associated with neuroinflammation and neuronal apoptosis [148]. Moreover, the TGF-β1-overexpressing mice exhibit AD-like cerebrovascular pathology, further validating the involvement of TGF-β1 in the pathogenesis of AD [148]. These observations imply the involvement of post-COVID-19 Guillain-Barré syndrome in AD, which needs to be studied further in detail. Besides the Guillain-Barré syndrome, psychiatric conditions after COVID-19 may also contribute to the development of AD. Animal studies have shown that PTSD-like induction accelerates the accumulation of Aβ through disrupting the corticotropin-releasing factor signaling, suggesting that the PTSD-like trauma can drive AD pathogenesis [149]. Similarly, depression-like stress also improves the progression of AD in rodents, most likely through elevating the levels of Aβ and phosphorylated tau [141, 142, 150].

Besides the aformentioned mechanisms, COVID-19 may also contribute to cognitive impairment in other ways such as cerebral ischemic damage, since vascular remodeling in infected regions has been observed in SARS-CoV-2-infected mouse brains independent of vascular infection [88]. Therefore, various studies have implied that COVID-19 can induce cognitive impairment, which may ultimately lead to the development of AD. The potential mechanisms include, but are not restricted to, the invasion of SARS-CoV-2 into the CNS, COVID-19-induced inflammation, long-term hospitalization and delirium, and post-COVID-19 syndrome. However, it will remain unknow whether AD is a long-term complication of COVID-19 untill solid evidence is obtained. More comprehensive investigations are needed to understand the contribution of COVID-19 to AD.

### Future work for clarifying the relationship between COVID-19 and AD

Although multiple studies have reported cognitive impairment in COVID-19 patients that suggests the potential roles of COVID-19 in AD development, there is still a lack of adequate evidence to support this relationship. The following studies may be necessary for clarifying this question.

First, short-term laboratory-based studies can be carried out immediately. For example, by using various AD animal models, the key AD pathological consequenes of SARS-CoV-2 infection can be examined, including cognitive functions, accumulation rate of amyloid plaque and NFT, and activation of immune cells in the brain, based on which a clue can be made on whether COVID-19 increases the risk of AD in populations with high-penetrant mutations of AD risk gene variants and whether it expedites the disease progression in AD patients. More excitingly, the hPSC-derived brain organoid models provide an excellent platform to mimick the situation of SARS-CoV-2 infection in the brains of AD patients [151]. By utilizing AD brain organoids, the effects of SARS-CoV-2 infection on the expression of AD risk genes and the degeneration of neurons can be determined, providing valuable information for the association of COVID-19 with AD.

Second, the COVID-19 patient samples including blood samples collected during hospitalization and post-mortem tissues provide a valuable source for research. To date, there have been very few studies examining the AD-related pathological changes in post-mortem tissues from COVID-19 patients, especially brain tissues. Furthermore, the sample sizes in these studies are very limited. These limitations have hindered our understanding of the potential causal effect of COVID-19 on AD. Therefore, studies with larger sample sizes and more specific classification (e.g., brain tissues from patients with both AD and COVID-19) are needed.

Third, it is essential to establish a long-term follow-up system for COVID-19 patients. With the huge number of confirmed cases of COVID-19, it is possible to build up large cohorts of COVID-19 patients, especially the aged patients. After long-term follow-up, many important retrospective studies could be carried out to demonstrate whether COVID-19 survivors have increased incidence of AD.

## The COVID-19 crisis negatively affects uninfected AD patients

The majority of AD patients are people living with dementia (PLwD) who rely on caregivers. Preventive strategies to combat COVID-19, such as isolation or quarantine, increase the burden of uninfected AD patients who need routine care and support. In this section, we discuss the effects of COVID-19 on uninfected AD patients or populations with AD risk.

### COVID-19 restrictions cause worsening of behavioral symptoms in AD patients

The COVID-19 epidemic has caused multiple social problems and concerns in AD patients without the viral infection. It has become obvious that the COVID-19-driven social isolation and quarantine have various adverse effects. People suffer from loneliness, depression and anxiety, and this situation is even worse for AD patients. Quarantine induces a rapid increase of behavioral and psychological symptoms in approximately 60% of patients with dementia including AD, FTD, dementia with Lewy bodies, and vascular dementia. In particular, AD patients have higher risk of anxiety than other types of dementia [152]. These behavioral problems are not restricted to people living in their own houses but also reported in AD patients who live in retirement homes, which are forced to physically isolate their residents.

Moreover, worsened depression and anxiety symptoms associated with the restrictions of COVID-19 have also been observed, similar to the situation of quarantine [153]. A survey in Argentina has reported worsening of behavioral symptoms in elderly subjects with dementia living in the community. About 60% of subjects with dementia during the epidemic displayed new onset of behavioral symptoms or exacerbation of pre-existing behavioral symptoms. Symptoms of anxiety, depression, and sleep disorders accounted for about 33%, 13% and 15% of the subjects, respectively [154]. The “stay-at-home” and visitation restrictions under COVID-19 save lives, at a cost of the mental health of PLwD and caregivers, as “non-essential” activities like psychosocial interventions are cut due to the safety concerns [155]. Therefore, negative consequences such as loneliness, agitation, depression, and caregiver burden may arise, and what to make things worse is that these people were left there to handle long-term mental health problems by themselves. Therefore, online services are urgently needed to continue routine interventions for PLwD.

### The COVID-19 pandemic has adverse effects on AD prevention

The isolation or contact restriction in the COVID-19 pandemic not only causes problems for AD patients, but also leads to adverse consequences for AD prevention.

Physical activity (PA) has been identified as a key factor in preventing AD through improving cerebral perfusion, facilitating neurogenesis and synaptogenesis, reducing neuronal loss, preserving brain volume in regions vulnerable to AD, and inhibiting Aβ accumulation and tau phosphorylation [156]. PA also significantly reduces the risk of myocardial infarction, stroke, and diabetes, which, in turn, diminishes AD morbidity [156]. Physical inactivity is assumed to account for one-third of the global prevalence of AD [157]. However, COVID-19 restrictions have curtailed PA globally, especially in the elderly [158]. A cross-sectional online survey suggests that there has been a 26.5% decrease of PA level in the older community in Japan. Similarly, a global decrease (mean 27.3% within 30 days following the declaration of a pandemic) of daily steps was reported in a descriptive study. These alarming results demonstrate that under the circumstances of the COVID-19 pandemic, new preventive approaches are needed that account for the suspected long-term lifestyle changes [158].

## Conclusions

SARS-CoV-2 has caused a global pandemic of COVID-19. Apart from attacking the respiratory system, current evidence suggests that SARS-CoV-2 is able to invade the CNS through hematogenous and neural routes and cause neurological problems that include cognitive impairment *via* viral infection, neuroinflammation, APP metabolism dysfunction, long-term hospitalization and delirium, post-COVID-19 syndrome, and other possible mechanisms [1]. On the other hand, AD has been indicated as one of the most common CNS comorbidities of COVID-19, imposing a greater burden on patients, society, and the economy. AD significantly increases COVID-19 morbidity and mortality due to aging, the AD-induced direct and indirect pathologic changes, drug-drug interactions, nutritional disorders, and the lack of self-care and cognitive abilities of AD patients. Moreover, the isolation or contact restriction in the COVID-19 pandemic also negatively affects uninfected AD patients and hinders AD prevention. Hence, all these findings suggest the importance to understand the underlying neurobiology of COVID-19, which needs to be clarified in future work.

Future studies on the relationship between COVID-19 and AD can be designed in the following aspects: (1) to identify the mechanisms of how SARS-CoV-2 invades the CNS and infects CNS cells; (2) to extensively investigate the reasons for increased COVID-19 mortality among AD patients and design corresponding therapeutic strategies; (3) to demonstrate the effects of COVID-19 on the AD-relevant pathological and behavioral changes using animal models and patient samples; (4) to establish long-term follow-up cohorts for analyzing the correlation between COVID-19 and AD; and (5) to develop new interventions that enable people to access required care under the likely long-lasting effects of COVID-19.

## Data Availability

Not applicable.

## Abbreviations

Aβ: Amyloid-beta
ACE2: Angiotensin converting enzyme 2
Ach: Acetylcholine
AD: Alzheimer’s disease
ApoE: Apolipoprotein E
APP: Amyloid precursor protein
ARDS: Acute respiratory distress syndrome
ChAT: Choline acetyltransferase
ChEI: Cholinesterase inhibitor
CMR: Case mortality rates
CNS: Central nervous system
COVID-19: Coronavirus disease 2019
DIC: Disseminated intravascular coagulation
FTD: Frontotemporal dementia
HHV-6A: Human herpesvirus 6A
hPSC: Human pluripotent stem cell
ICU: Intensive care unit
IL-1: Interleukin-1
IL-6: Interleukin-6
MERS-CoV: Middle East respiratory syndrome coronavirus
MSOF: Multi-system organ failure
NFT: Neurofibrillary tangle
NLRP3: NOD-, LRR- and pyrin domain-containing protein 3
Nsp: Non-structural proteins
PA: Physical activity
PLwD: People living with dementia
PTSD: Post-traumatic stress disorder
RBD: Receptor binding domain
RdRp: RNA-dependent RNA polymerase
SARS-CoV: Severe acute respiratory syndrome coronavirus
SARS-CoV-2: Severe acute respiratory syndrome coronavirus-2
S protein: Spike protein
TGF-β1: Transforming growth factor-β1
TMPRSS2: Transmembrane protease serine 2
TNF-α: Tumor necrosis factor-alpha
WHO: World health Organization
